# Modelling malaria incidence with environmental dependency in a locality of Sudanese savannah area, Mali

**DOI:** 10.1186/1475-2875-8-61

**Published:** 2009-04-10

**Authors:** Jean Gaudart, Ousmane Touré, Nadine Dessay, A lassane Dicko, Stéphane Ranque, Loic Forest, Jacques Demongeot, Ogobara K Doumbo

**Affiliations:** 1 Biostatistics Research Unit, Laboratory of Education and Research in Medical Information Processing (LERTIM), EA 3283 Aix-Marseille University, Faculty of Medicine, 27 Bd Jean Moulin, 13385 Marseille cedex 5, France; 2 Malaria Research and Training Centre (MRTC), Department of Epidemiology of Parasitic Diseases, Faculty of Medicine, Pharmacy and Odonto-Stomatology, University of Bamako, Mali, BP 1805 Bamako, Mali; 3 Laboratory of Hydrology Transfers and Environment (LTHE), Domaine Universitaire, 38400 Saint Martin d'Hères, France; 4 Laboratory of Parasitology-Mycology, Hôpital de La Timone, AP-HM, 13005 Marseille, France; 5 INSA Rouen, Laboratory of Mathematics and informatics EA3226, Place Emile Blondel, BP 08, 76131 Mont Saint-Aignan, France; 6 University Joseph Fourier Grenoble, Laboratory of Techniques for Imaging, Modelling and Complexity – Informatics, Mathematics and Applications Grenoble, TIMC-IMAG UMR NRS 5525, Faculty of Medicine, Domaine de la Merci, 38710 La Tronche, France

## Abstract

**Background:**

The risk of *Plasmodium falciparum *infection is variable over space and time and this variability is related to environmental variability. Environmental factors affect the biological cycle of both vector and parasite. Despite this strong relationship, environmental effects have rarely been included in malaria transmission models.

Remote sensing data on environment were incorporated into a temporal model of the transmission, to forecast the evolution of malaria epidemiology, in a locality of Sudanese savannah area.

**Methods:**

A dynamic cohort was constituted in June 1996 and followed up until June 2001 in the locality of Bancoumana, Mali. The 15-day composite vegetation index (NDVI), issued from satellite imagery series (NOAA) from July 1981 to December 2006, was used as remote sensing data.

The statistical relationship between NDVI and incidence of *P. falciparum *infection was assessed by ARIMA analysis. ROC analysis provided an NDVI value for the prediction of an increase in incidence of parasitaemia.

Malaria transmission was modelled using an SIRS-type model, adapted to Bancoumana's data. Environmental factors influenced vector mortality and aggressiveness, as well as length of the gonotrophic cycle. NDVI observations from 1981 to 2001 were used for the simulation of the extrinsic variable of a hidden Markov chain model. Observations from 2002 to 2006 served as external validation.

**Results:**

The seasonal pattern of *P. falciparum *incidence was significantly explained by NDVI, with a delay of 15 days (p = 0.001). An NDVI threshold of 0.361 (p = 0.007) provided a Diagnostic Odd Ratio (DOR) of 2.64 (CI95% [1.26;5.52]).

The deterministic transmission model, with stochastic environmental factor, predicted an endemo-epidemic pattern of malaria infection. The incidences of parasitaemia were adequately modelled, using the observed NDVI as well as the NDVI simulations. Transmission pattern have been modelled and observed values were adequately predicted. The error parameters have shown the smallest values for a monthly model of environmental changes.

**Conclusion:**

Remote-sensed data were coupled with field study data in order to drive a malaria transmission model. Several studies have shown that the NDVI presents significant correlations with climate variables, such as precipitations particularly in Sudanese savannah environments. Non-linear model combining environmental variables, predisposition factors and transmission pattern can be used for community level risk evaluation.

## Background

Malaria kills between 1.1 and 2.7 million people per year, including almost one million children under the age of five years in sub-Saharan Africa [1,2]. The methods of control recommended by the WHO are based not only on chemical and physicochemical control and prophylaxis but also on environmental measures (e.g. draining of backwaters), targeted means of prevention and early detection of epidemics. The risk of *Plasmodium falciparum *infection is variable over space and time [3,4], and this variability is related to environmental and climatic changes [5]. The specific management of an environment favouring the proliferation of vectors (*Anopheles*) can significantly decrease transmission [6]. The choice of interventions and their relative importance are determined by the knowledge of environmental heterogeneity [3,4,6].

Climatic and environmental factors affect *Anopheles *production, survival, speed of reproduction and parasitic life cycle [7-17]. This relationship explains the distribution of *P. falciparum*. Rainfall and temperature play a major role, directly on *Anopheles *behaviour or indirectly on breeding sites. Vegetation is also an environmental factor depending on climatic evolutions, which influences the behaviour of the vector directly and indirectly [18]. In regions with alternate dry and rainy seasons, the transmission of malaria is seasonal, epidemic or endemo-epidemic. The principal parameters influenced by rainfall and temperature are aggressiveness (depending on *Anopheles *density and on the length of their gonotrophic cycle), contagiousness and *Anopheles *mortality. The variation is highly structured across geographic and temporal sub-populations. The high diversity during the rainy season, when transmission rate peaks, contrasts with the low diversity during the dry season, when both mosquito population size and malaria transmission rate are low.

Following the first descriptions of the parasite and its life cycle, mathematical models have been designed by Ronald Ross (1909). These models not only brought a better understanding of the transmission, but also improved the first vectorial control strategies [19-21]. The differential equations of Ross were modified by MacDonald. Other authors introduced additional concepts such as multiple infection, immunity, co-infection [for example, see [22-24]]. In these historical models, parameters of transmission were constant, even if vectorial behaviour presents temporal evolution [9,23]. Despite the strong relationship between malaria risk and environmental factors [8,9,11], environmental effects have rarely been included in malaria transmission models, probably because of technical difficulties in obtaining environmental data from field. Satellite imagery has been used to investigate covariates related to disease transmission, particularly NDVI (Normalized Difference Vegetation Index) [25-31]. Indeed, satellites from the NOAA series (National Oceanic and Atmospheric Administration) provide a vegetation survey at the climatic scale. These NOAA data have shown their usefulness in the monitoring of vegetation [32-39]. Furthermore, NOAA data are freely available, and provide good information on environmental field characteristics. The relationship between NDVI data and malaria incidence has been demonstrated, and thus, NDVI can be used as a proxy of climatic and environmental factors [18,28,29,40].

Incorporating remotely sensed information on environment into a transmission model can improve the knowledge of the epidemiological pattern of malaria. A micro-epidemiology analysis, pivotal for testing control measures or individual risk factors and for forecasting epidemiological pattern of malaria, has to integrate environmental factors.

The aim of this study was to provide a temporal model of malaria transmission, based on classical models and adapted to field data (Sudanese savannah area), with environmental dependency introduced by NDVI simulations.

## Methods

### Parasitological data

**Figure 1:**
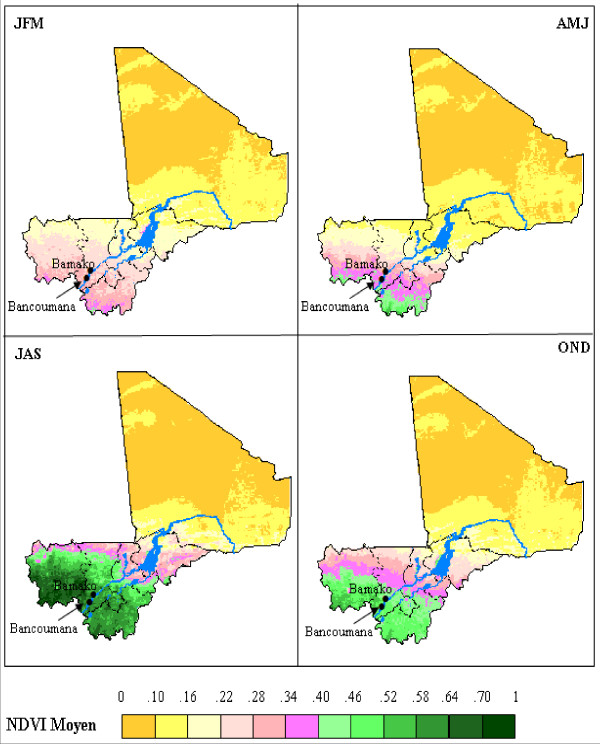
**Maps of Mali showing NDVI means**. The coloured scale shows the NDVI means estimated over the four trimesters, 1982–2006.

Data was obtained by a field study in the locality of Bancoumana, located in the Sudanese savannah zone of the Upper Niger valley (district of Kati) about 60 km south-west of Bamako, the capital of Mali (Figure 1). This locality covers an area of 2.5 km^2 ^and has a population of 8,000 inhabitants. A dynamic cohort was constituted in June 1996 and followed up until June 2001. The study included 173 of the 340 households, selected at random from each of the four geographic blocks of the village, using a stratified sampling. In each household, all children aged 0 to 12 years were followed up, constituting the dynamic cohort (for more information, see [5]). The surveys [22] were carried out at the rate of about one survey every two months during the rainy season and one every three months during the dry season. The intervals between surveys were defined on the basis of the previous knowledge of the seasonal transmission [41,42]. For each survey, a blood sample was taken and parasitaemia assessed. A trained team of biologists carried out microscopy to search for *P. falciparum *and its gametocytes in Giemsa-stained thick blood films. Biological diagnostic was subjected to quality control. Infection was defined as the presence of the parasite in the thick blood film. The time series of incidences of *P. falciparum *parasitaemia and gametocytaemia were analysed in the present work in order to provide a dynamical model of malaria transmission.

Community permission and individual Informed Consent were obtained according to the stepwise process described by Diallo *et al *[43].

### Remote sensing

For remotely sensed data the 15-day composite NDVI provided by the GIMMS group (Global Inventory Monitoring and Modelling Studies) at NASA/GSFC (National Aeronautics and Space Administration/Goddard Space Flight Center) was used (Figure 1). NDVI was derived from channels 1 and 2 of the NOAA AVHRR (Advanced Very High Resolution Radiometer) satellite series 7, 9, 11, 14, 16 and 17. NDVI data were acquired over 25 years from July 1981 to December 2006. Data obtained from the focus on Bancoumana were used into the model transmission.

NDVI was calculated as the normalized difference of corrected reflectance of the NIR (near infrared ranged from 0.725–1.10 μm) and visible (ranged from 0.58–0.68 μm) channels using AVHRR GAC (Global Area Coverage, 4 km resolution) data. The 15-day composites were generated by selecting the maximum value of NDVI, in order to minimize contamination by clouds. Spatial resolution was re-sampled to 8 km × 8 km pixels. The NDVI GIMMS data set was improved using the navigation procedure provided by El Saleous *et al *[44], the calibration of visible and NIR channels [45]. The solar zenith angle values from AVHRR sensor were also corrected [46]. Effects of stratospheric aerosols due to volcanic eruptions of El Chichon (1981) and Mount Pinatubo (1991), during April 82-December 84 and June 91-December 93, have been corrected using the method developed by Vermote *et al *[47]. No correction has been applied to correct for atmospheric effects due to water vapour, Rayleigh scattering or stratospheric ozone.

An additional Quality Control was applied to the NDVI data set to filter unrealistic values (*i.e. *values larger than 1 or smaller than -1). NDVI values retrieved from spline interpolation or average seasonal profile have been considered as missing data. For each fortnight, data were calculated using the maximum NDVI value of 15-day composites, in order to provide time series of vegetation characteristics, in the locality of Bancoumana.

### Statistical analyses

The statistical relationship between NDVI and incidence of *P. falciparum *infection was assessed by classical ARIMA time series analysis [48,49] after logarithmic transformation of the incidences. These established statistical models have been used to model time series, by breakdown into tendency, cyclic and accidental components, and also to identify significant predictor [50]. Observed NDVI was introduced in the ARIMA analysis as a covariate and tested, and temporal delays were also analysed.

ROC (Receiver Operating Characteristic) analysis was used to determine an NDVI threshold predicting an increase in the parasitaemia incidence. The quality of this threshold was assessed by AUC test (Area Under the ROC Curve) and by the DOR (Diagnostic Odd Ratio) [see for example [51,52]]. Statistical analysis was performed using SPSS 15.0^® ^(SPSS Inc., Chicago, Ill., USA). A significance level of α = 0.05 was used for hypothesis tests.

### Malaria model

**Figure 2:**
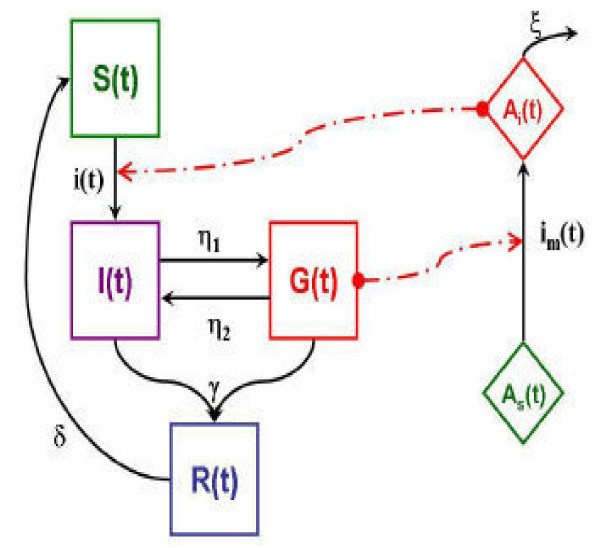
**Malaria transmission model adapted to Bancoumana's data**. *S*: susceptible state, *I*: infected not contagious state, *G*: contagious state, *R*: resistant state. *A*_*s*_: susceptible state (*Anopheles*), *A*_*i*_: contagious state (*Anopheles*).

Malaria transmission was modelled using a deterministic approach. A SIRS-type model [19,23] was adapted to Bancoumana's data. The model was built on the MacDonald equations, specifying states for infected-not-contagious and contagious children (such as Bailey's model [20]) and adding a resistant state (such as Dutertre's model [23]). The first state *S *was defined as the proportion of susceptible children. The second state *I *represented the proportion of infected but not contagious children, *i.e. *children without gametocytaemia. The third state *G *represented the production of contagious children, *i.e. *children with gametocytaemia. Indeed, the transmission needs two parasitic cycles, an asexual cycle in human and a sexual cycle in *Anopheles*, this latter is made possible by gametocyte production in human. The last state *R *represented the proportion of children "resistant" to infection, *i.e. *children were considered as resistant during the effectiveness of curative treatment (Figure 2). The transition from state *S *to state *I *depended on vectorial and climatic factors (**i(t)**), and children were considered without effective immunity. Demographic factors as human natality and mortality have been neglected. Infected but not contagious children (state *I*) could become contagious with a parameter **η**_**1 **_(production of gametocytes) or resistant with a parameter **γ **(curative treatment). Contagious children (state *G*) could loose their contagiousness with a parameter **η**_**2**_, or could become resistant (with the parameter **γ**). The parameter **δ **represented the inverse of the duration of the treatment effectiveness.

The vectorial part of the cycle was modelled with a two-state model: the state of susceptible *Anopheles *(*A*_*s*_) and the state of contagious *Anopheles *(*A*_*i*_). The transition took place when susceptible *Anopheles *had a blood meal on contagious children (*G*), with a parameter **i**_**m**_**(t) **depending on vectorial and climatic factors. Vectorial parameters were density (**μ**), length of the gonotrophic cycle (**ν**), contagiousness (**β**), aggressiveness (**α**), and mortality (**ξ**). Human contagiousness (**ζ**) has been added to the model. Model equations have been written as follows:

(a)

(b)

(c)

(d)

(e)

(f)

(g)

where ***VI*(t) **was the vegetation index (NDVI) and represented environmental factor modelling. Environmental factors influenced vector mortality **ξ**, length **ν **of the gonotrophic cycle, vectorial aggressiveness **α**, with a time lag **θ**.

Parameter estimations were issued from a review of published works [53]. The parameter values have been bounded within the range of published estimations and have to minimize quality indexes (RMSE and MAPE, see later). Furthermore, these values were validated by senior entomologists and parasitologists. Initial conditions were estimated from observed data (June 1996) (Table 1). *Anopheles *mortality and NDVI values were related by a functional form modelling a slow decrease of mortality when NDVI increased. This relationship provided also a high mortality constant rate for the lowest values of NDVI (during dry season), below a constant threshold (**τ**). The addition of 1 to the denominator permitted to avoid null values. χ represented the indicator function:

**Table 1:** Parameter estimations and initial conditions.

**Parameters**	**Definitions**	**Estimations**
***α***	number of bites per *Anopheles *per night	0.27

***β***	probability of a susceptible human becoming infected after one single infected bite	0.1

***γ***	probability of becoming resistant after being infected or contagious	0.01

***δ***	probability of becoming susceptible after being resistant	0.04

***ζ***	probability of a susceptible *Anopheles *becoming infected after one single bite on a contagious human	0.01

***ξ***	basic *Anopheles *mortality per day *	0.014

***μ***	basic *Anopheles *density *	1

***η 1***	probability of acquiring contagiousness	0.05

***η2***	probability of loosing contagiousness	0.75

***ν***	basic length of gonotrophic cycle *	0.1

***θ***	Time lag of NDVI influence (days)	15

***τ***	lowest value of the NDVI influencing *Anopheles *behaviour	0.25

***S(0)***	initial proportion of susceptible human	0.45

***I(0)***	initial proportion of infected not contagious human	0.5

***G(0)***	initial proportion of contagious human	0.05

***R(0)***	initial proportion of resistant human	0

* these basic values were modified by NDVI.



The transmission rates (**i(t) **and **i**_**m**_**(t)**) were also related to NDVI values by a functional form modelling the increase of transmission when NDVI increased and low transmission constant rate during dry season.

The basic reproductive number **z**_**0 **_has been calculated from these equations:



Note that the basic reproductive number was null for low values of NDVI. No transmission could occur if climatic factors do not favour the normal behaviour of *Anopheles*.

### Environmental model

Environmental factors were considered as an extrinsic variable of the Bancoumana's model. Thus, these factors have been independently modelled. Among the environmental factors related to malaria, observations from 1981 to 2001 were analysed. The extrinsic NDVI variable ***VI*(t) **was simulated using a hidden Markov chain model. Observations from 2002 to 2006 served as external validation.

**Figure 3:**
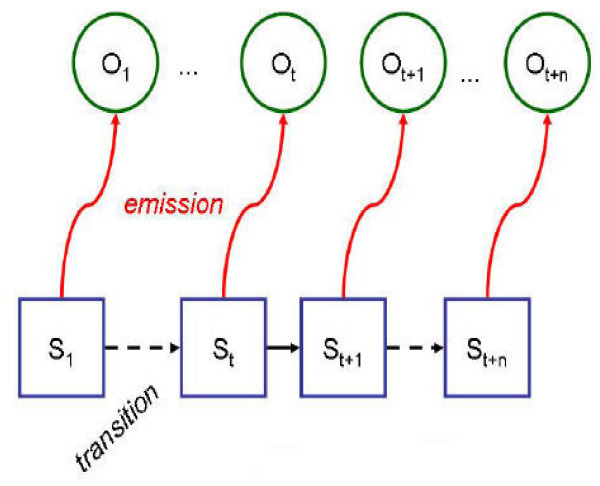
**Hidden Markov model**. The blue squares represent the time sequence of hidden classes (states). The green circles represent the time sequence of observed data.

Hidden Markov models (HMM) were introduced by Baum and Petrie at the end of the 60's [54,55]. This family of stochastic models has been then developed both theoretically (for example [56-58]) and in terms of applications particularly in hydrology and climatology sciences [59-61]. These methods make the assumption that the observed data are generated by an underlying finite mixture of distributions, itself organized in a Markov chain (Figure 3). Used for sequence analysis, they provide a classification model of sequence parts. Indeed, the hidden variable can be interpreted as a class of the observed variables.

The hidden Markov model {(*S*_k_, *O*_k_)} is constituted by a set of finite states *S*_k_, k ∈ {1, K}, associated to a probability distribution. Discrete time transitions between these hidden states are provided by transition probabilities, and the resulting time sequence of states (*S*_*t*_, *t *> 0) is a homogeneous Markov chain of recursivity order 1:



At time t, for a given state *S*_t _= k, an observation *O*_t _= o is issued following the probability distribution associated to this state, the emission probability p(*O*_t _= o/*S*_t _= k). Then, the sequence of observations (*O*_t_, t > 0) is a sequence of random variables conditionally independent, given the sequence of hidden states.

Such a model is defined by:

• p(*S*_t = 1 _= k)_k ∈ {1, ..., K}_, initial probabilities (at time t = 1)

• p(*S*_t+1 _= j/*S*_t _= i)_(i, j) ∈ {1, ..., K}_^2^, ∀ t, elements of the matrix of transition probabilities

• p(*O*_t _= o/*S*_t _= k)_k ∈ {1, ..., K}_, emission probabilities

Following this approach, a HMM of NDVI was designed, where the hidden states represented the monthly evolution of climate and environment. An emission probability represented the probability that an NDVI value occurred at a time t, given the environment of a determinate month. A transition probability represented the probability of an environmental change. The EM algorithm was used for the estimation of emission and transition probabilities and then simulating NDVI. The choice of the hidden states (1 state representing each month, 2 months or one season) was conducted by the quality indexes (RMSE and MAPE see below).

### Quality assessment and implementation

Quality of the predictions was performed using the root mean squared error (RMSE) and the mean absolute percentage error (MAPE) defined as follows:





where h was the time-lag of prediction,  the prediction at time t, and *X*_t _the observed value at time t.

The complete model was implemented using Matlab 7.0.4 ^®^, (Mathworks, Inc., Natick Massachusetts, USA)

## Results

### Time series analysis

**Figure 4:**
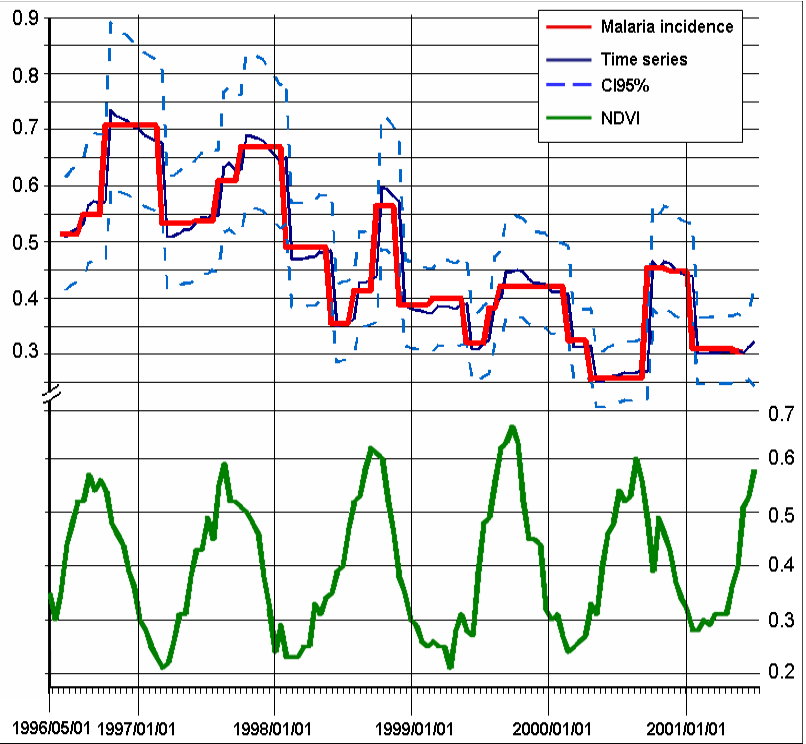
**Incidence of *P. falciparum *and NDVI**. X-axis: time (fortnight); Y-axis: NDVI (left) or Incidence (right). The time-series model (modelling *P. falciparum *incidence by NDVI and a constant decrease in incidence) is presented in blue (bold). The bounds of the 95% confidence interval are indicated as dotted lines. The observed incidences are presented in red (bold) and NDVI values in green.

The seasonal pattern of *P. falciparum *incidence was significantly explained by NDVI (Figure 4), with a delay of 15 days (p = 0.001). The value of the adjusted R2 (R_adj_2 = 89%) was relatively high, and the quality indexes were relatively low (RMSE = 0.04, MAPE = 5.61). Thus, the statistical model, using NDVI as covariate, showed a satisfactory goodness-of-fit. The known decrease in infection from year to year was significant (p = 0.001), but remained weak (-0.109 after logarithmic transformation, standard deviation SD = 0.031).

### NDVI threshold

The NDVI values observed around Bancoumana were less than 0.34 during the dry season and the highest values (>0.52) have been observed during the rainy season. The ROC analysis has provided an NDVI threshold of 0.361. Beyond this threshold, the odd ratio of an increase in the parasitaemia incidence was significant, estimated at DOR = 2.64 (CI95% [1.26;5.52]).

**Figure 5:**
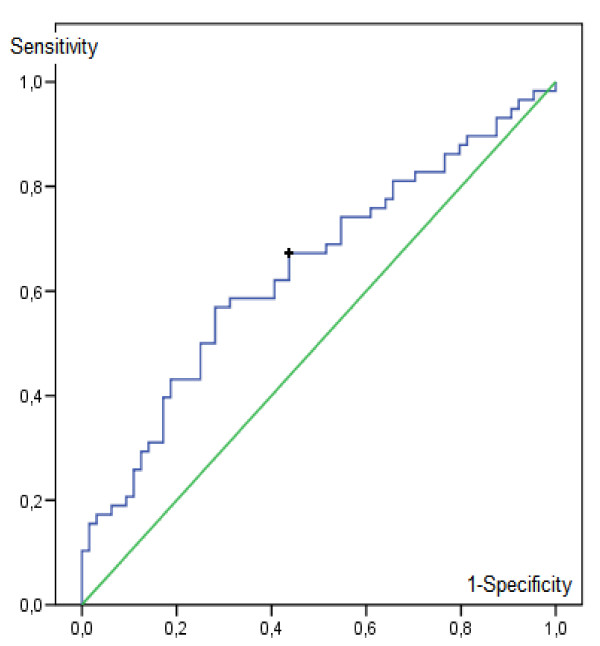
**ROC curve of NDVI for the prediction of an increase in parasitaemia incidence (blue)**. The green line represents the first bisector. The black cross represents the NDVI threshold with the best DOR (NDVI = 0.361; sensitivity of 67%; specificity of 56%).

The area under the ROC curve was 0.65 (CI95% [0.54;0.74]), significantly different from 0.5 (p = 0.007) (Figure 5).

### NDVI simulations

The probabilities of changes in environmental characteristics (the transition probabilities from one month to another) were null if these 2 months were not contiguous. Indeed, environmental characteristics cannot change suddenly. Persistence of environment was also possible. Indeed, environmental characteristics may persist from one month to the next, with a probability of 50%. An environmental change from one month to the next was also possible, with a probability of 50%. These transmission probability were constant, whatever the months were. These estimations reflected the seasonal nature of the phenomenon: persistence of environmental characteristics between 2 contiguous months or progressive changes.

**Figure 6:**
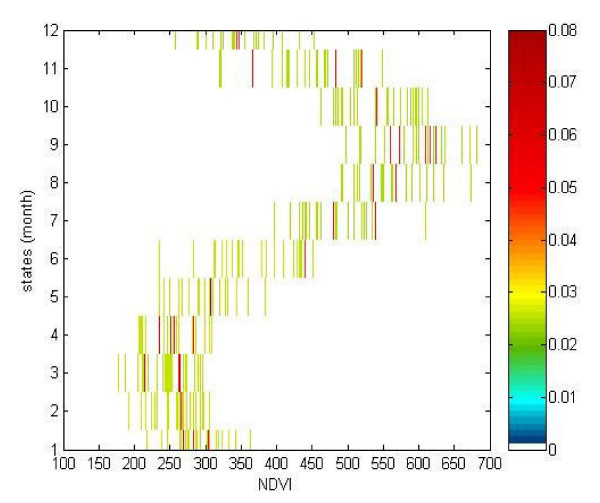
**Emission probabilities**. The coloured scale shows the probability that a given NDVI (x-axis, ×1000) occurs for a given month (y-axis).

Probabilities of observing specific values of NDVI, the emission probabilities estimated for each month (Figure 6), were not high and reflected also the seasonal changes in NDVI regimes. Indeed, the probabilities that high NDVI values occur in January, February or March were nil and small NDVI values could occur with non-zero probabilities; for example there was 35.0% of chance observing an NDVI between 0.2 and 0.25 during March (cumulative probability). *A contrario*, the probabilities that high NDVI values occur were important during August and September; for example there was about 47.6% of chance observing an NDVI between 0.6 and 0.65 during September (cumulative probability).

The choice of hidden classes reflecting the monthly scale of seasonal changes was conducted by MAPE and RMSE (Table 2) between predictions and validation set values (2002–2006 NDVI).

**Table 2:** Choice of hidden classes. Mean absolute percentage error (MAPE) and root mean squared error (RMSE) by external validation (2002–2006 NDVI).

Hidden classes	MAPE	RMSE
	
Seasons	0.1992	123.8637
2 months	0.1981	119.8227
1 month	0.1777	59.6326
15 days	0.1849	121.9749

**Figure 7:**
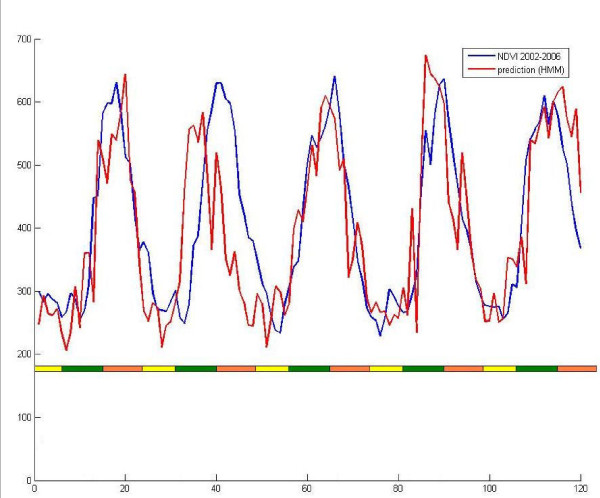
**NDVI simulation**. X-axis: number of fortnight; Y-axis: NDVI. The red line represents the prediction made by hidden Markov model. The blue line represents the observed NDVI from 2002 to 2006 (external set). The colored scale shows the three seasons, rainy (green), cool and dry (orange) and warm and dry (yellow).

The external validation showed the smallest values of MAPE (0.178) and RMSE of (59.63) for a monthly scale of seasonal changes. The model predicted adequately seasonal variations (Figure 7) and then could be used as environmental factor for malaria modelling.

### Malaria model

**Figure 8:**
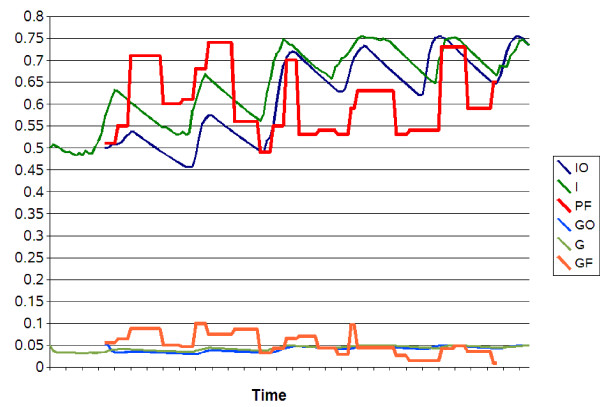
**Observed parasitaemia and gametocytaemia incidences versus predicted values**. X-axis: time (10 days). Y-axis: incidences. The red and orange lines represent the observed incidences of respectively *P. falciparum *parasitaemia (PF) and gametocytaemia (GF), after removing the trend of the time series. The blue lines (dark and light blue) represent the predicted values of respectively parasitaemia (IO) and gametocytaemia (GO) incidences, using observed NDVI. The green lines (dark and light green) represent the predicted values of respectively parasitaemia (I) and gametocytaemia (G) incidences, using HMM model of NDVI.

**Figure 9:**
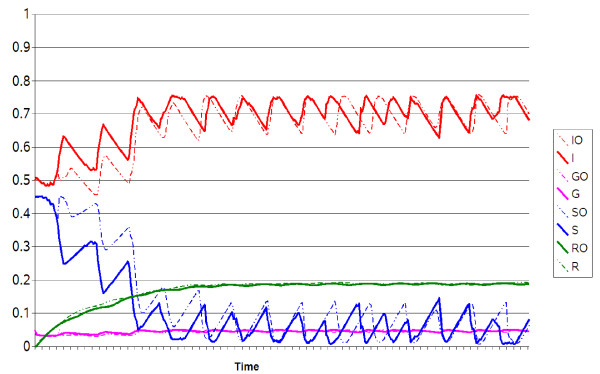
**Prediction of malaria incidence**. X-axis: time (15-days). Y-axis: incidences. The solid or dotted lines represent the predicted values using respectively HMM model or observed NDVI. I: predicted incidence of *P. falciparum *parasitaemia using HMM model. IO: predicted incidence of *P. falciparum *parasitaemia using observed NDVI. G: predicted incidence of *P. falciparum *gametocytaemia using HMM model. GO: predicted incidence of *P. falciparum *gametocytaemia using observed NDVI. S: predicted incidence of susceptible children using HMM model. SO: predicted incidence of susceptible children using observed NDVI. R: predicted incidence of resistant children using HMM model. RO: predicted incidence of resistant children using observed NDVI.

The deterministic transmission model, with stochastic environmental factor, predicted an endemo-epidemic pattern of malaria infection. Indeed, incidences of parasitaemia fluctuated around 70 per 100 inhabitants per 15-days. The model provided a seasonality pattern of incidences, with low values for the dry seasons (about 65%) and high values for the rainy seasons (75%). These oscillations of predicted incidences were similar to observed values (Figure 8 and 9). Quality indexes have shown the smallest values (MAPE = 0.07, RMSE = 0.01 for parasitaemia) for a monthly model of environmental changes (Table 3). Incidences of gametocytaemia fluctuated around 5 per 100 inhabitants per 15-days. Oscillations of predicted gametocytaemia incidences were less pronounced as observed incidences, but indexes have also shown low values (MAPE = 0.01, RMSE = 0.001 for gametocytaemia).

**Table 3:** Quality assessment of malaria transmission model for different hidden classes.

**Hidden classes**	**Parasitaemia**		**Gametocytaemia**	
	MAPE	RMSE	MAPE	RMSE
	
Season	0.082	0.0133	0.0118	0.0015
2 months	0.0816	0.0132	0.0122	0.0015
1 month	0.0719	0.0121	0.0117	0.0014
15 days	0.729	0.0123	0.0114	0.0014

Mean absolute percentage error (MAPE) and root mean squared error (RMSE) between prediction and observed incidences are presented for P. falciparum parasitaemia and gametocytaemia.

Incidences of parasitaemia and gametocytaemia were adequately modelled, using the observed NDVI as well as the HMM model of NDVI (Figure 8 and 9). Indeed, seasonal variations and mean value of incidences were similar using both NDVI data.

## Discussion

In this study, a malaria transmission model was designed, using NDVI as a proxy of environmental factors, especially humidity conditions. The NDVI allows linking detected physical characteristics of plants with their functional status and monitoring their temporal evolution. It helps to extract a strong signal related to vegetation and provides good contrast with other earth's surface objects [62]. Several studies have shown that the NDVI presents significant correlations with climate variables such as precipitations and land surface temperatures [63-65], particularly in Sudanese savannah environments. Thus, NDVI can be used when climatic data as well as hydrological or environmental field characteristics are not easily available. The relationship between NDVI and malaria epidemiology is well known and is mostly due to the climatic dependency of vector behaviour [17,25-27,30,66,67]. Indeed, it has been suggested that the number of breeding sites and NDVI values increase with the soil moisture state, the latter being multi-factorial [26,27]. Furthermore, the 15-days lag between NDVI and malaria incidence has also been reported in other studies [18,27,68]. The NDVI threshold deduced from this study is consistent with other publications where an NDVI between 0.35 and 0.4 is associated with an increased incidence [25,28].

Note that a clear relationship between NDVI and malaria has been shown in sahelian or Sudanese savannah environments (such as Bancoumana's region), but not in other regions [41], characterized by an absence of seasonality or persistent moisture (for example rice-field, flood regions).

It is clear that the use of observed NDVI allows adequate predictions of parasitaemia incidences. However, NDVI data are not always available. It is then necessary to use an adequate predictive model. The HMM model brings explanatory structures, such as seasonal classes represented by hidden classes. The stochasticity of the phenomenon is also modelled by HMM. In such an epidemiological model, stochastic events can lead to crossing a threshold and to an epidemiologic amplification. Because of this stochastic nature of modelling (Figure 9), a temporary gap has been found between the model using observed NDVI data and the model using NDVI data simulated by HMM. Other models (including sinusoidal models [18,53]) do not take into account this stochastic nature of natural phenomena. Other stochastic models can be used, for example non-parametric regressions [53], but these models allow rarely also an explanatory approach.

Based on historical models, this designed model reflects the non-linearity of epidemiological phenomenon (in contrast to other approaches [18,68]). This model respects the chronological order of appearance of gametocytes, which has not been the case with other historical models [19,20,23], but is a key point for malaria transmission. The proposed basic reproductive number has the same form as that of MacDonald. The values of RMSE and MAPE are relatively low, both for parasitaemia and for gametocytaemia.

As the field study has included only children, the relative immunity was considered as inefficient here. In addition, since infected children have been treated, they were considered as "resistant" for the duration of the effectiveness of treatment. The collection method did not change over the study period. Cases of malaria have been confirmed biologically, biological diagnosis was subjected to continuous quality control [5]. The observed decreasing trend (and even the trend estimated with the ARIMA statistical analysis) in the incidence of *P. falciparum *is not taken into account by the deterministic model. This trend has already been observed in other field studies on the same site [41,42]. It is unlikely that this trend was due to the natural evolution of malaria in this region. The NDVI values observed during that period exclude climate change. There have been no further developments in the village, neither as regards the number of people nor about known risk factors (breeding site control for example). Most probably, this decreasing trend in the incidence of *P. falciparum *was linked to the presence of the medical team in the village.

## Conclusion

In this study, remote-sensed data were coupled with field study data in order to drive a malaria transmission model. In a micro-epidemiology context, NDVI provided useful variables, improving malaria transmission modelling. Non-linear model combining environmental variables, predisposition factors and transmission evolution can be used for community level risk evaluation. Accumulating data [47,66] point to the need of integrating several control measures to enhance efficiency. Thus, control programmes, such as vector control, impregnated net use or early detection and treatment, should to be tailored to environmental conditions.

## List of abbreviations

ARIMA: Autoregressive Integrated Moving Average; AUC: Area Under the Curve; AVHRR: Advanced Very High Resolution Radiometer; DOR: Diagnostic Odd Ratio; GAC: Global Area Coverage; GIMMS: Global Inventory Monitoring and Modelling Studies; GPS: Global Positioning System; GSFC: Goddard Space Flight Center; HMM: Hidden Markov chain Model; MAPE: Mean Absolute Percentage Error; NASA: National Aeronautics and Space Administration; NDVI: Normalized Difference Vegetation Index; NIR: Near Infra Red; NOAA: National Oceanic and Atmospheric Administration; RMSE: Root Mean Squared Error; ROC: Receiver Operating Characteristic; WHO: World Health Organization.

## Competing interests

The authors declare that they have no competing interests.

## Authors' contributions

JG performed the statistical analysis and the mathematical model, drafted the manuscript and participated in the interpretation of data. OT performed the GPS/GIS data collection, the data computing and the validation in the field site of Bancoumana. He participated in the clinical, biological data collection. ND performed the NDVI extraction, and participated in the interpretation of results and drafted the manuscript. AD participated in the clinical, biological data collection in the field site of Bancoumana. He participated in the GPS/GIS data collection, the data computing and the validation. SR participated in the GPS/GIS data collection and validation. LS participated in the mathematical model and correction of the manuscript. JD supervised the statistical analysis and the mathematical modelling. He participated in the result interpretation and corrected the manuscript. OKD the PI of the Mali-Tulane TMRC led the team who conceived and designed the studies, and supervised the field work. He participated in the community consent protocol, in data collection, data monitoring, QA/QC of the data, data analysis and correction of the manuscript. All authors read and approved the final manuscript.
